# Assessment of Thyroid Carcinogenic Risk and Safety Profile of GLP1-RA Semaglutide (Ozempic) Therapy for Diabetes Mellitus and Obesity: A Systematic Literature Review

**DOI:** 10.3390/ijms25084346

**Published:** 2024-04-15

**Authors:** Catalin Vladut Ionut Feier, Razvan Constantin Vonica, Alaviana Monique Faur, Diana Raluca Streinu, Calin Muntean

**Affiliations:** 1 First Discipline of Surgery, Department X-Surgery, “Victor Babes” University of Medicine and Pharmacy, 2 E. Murgu Sq., 300041 Timisoara, Romania; catalin.feier@umft.ro; 2 First Surgery Clinic, “Pius Brinzeu” Clinical Emergency Hospital, 300723 Timisoara, Romania; 3 Preclinical Department, Discipline of Physiology, Faculty of General Medicine, “Lucian Blaga” University of Sibiu, 550169 Sibiu, Romania; 4 Faculty of Medicine, “Victor Babes” University of Medicine and Pharmacy, 2 E. Murgu Sq., 300041 Timisoara, Romania; alaviana.faur@student.umft.ro; 5 Department of Doctoral Studies, “Victor Babes” University of Medicine and Pharmacy, 2 E. Murgu Sq., 300041 Timisoara, Romania; diana.streinu@umft.ro; 6 Medical Informatics and Biostatistics, Department III-Functional Sciences, “Victor Babes” University of Medicine and Pharmacy, 2 E. Murgu Sq., 300041 Timisoara, Romania; cmuntean@umft.ro

**Keywords:** semaglutide, Ozempic, thyroid cancer, cancer risk, oncology

## Abstract

The broadening application of glucagon-like peptide (GLP)-1 receptor agonists, specifically semaglutide (Ozempic) for the management of diabetes and obesity brings a critical need to evaluate its safety profile, considering estimates of up to 20 million prescriptions per year in the US until 2035. This systematic review aims to assess the incidence of thyroid cancer and detail the spectrum of adverse events associated with semaglutide, focusing on its implications for patient care. Through a systematic search of PubMed, Scopus, and Embase databases up to December 2023, ten randomized controlled trials (RCTs) involving 14,550 participants, with 7830 receiving semaglutide, were analyzed, with an additional number of 18 studies that were separately discussed because they reported data from the same RCTs. The review focused on thyroid cancer incidence, gastrointestinal symptoms, and other significant adverse events attributed to semaglutide. The incidence of thyroid cancer in semaglutide-treated patients was less than 1%, suggesting no significant risk. Adverse events were predominantly gastrointestinal, including nausea (2.05% to 19.95%) and diarrhea (1.4% to 13%). Nasopharyngitis and vomiting were also notable, with mean prevalences of 8.23% and 5.97%, respectively. Other adverse events included increased lipase levels (mean of 6.5%), headaches (mean prevalence of 7.92%), decreased appetite (reported consistently at 7%), influenza symptoms (mean prevalence of 5.23%), dyspepsia (mean prevalence of 5.18%), and constipation (mean prevalence of 6.91%). Serious adverse events varied from 7% to 25.2%, highlighting the need for vigilant patient monitoring. These findings underscore the gastrointestinal nature of semaglutide’s adverse events, which, while prevalent, did not significantly deter from its clinical benefits in the treatment landscape. This systematic review provides a comprehensive assessment of semaglutide’s safety profile, with a focus on gastrointestinal adverse events and a low incidence of thyroid cancer. Despite the prevalence of gastrointestinal symptoms, semaglutide remains an efficacious option for managing diabetes and obesity. The detailed characterization of adverse events underscores the importance of monitoring and managing these effects in clinical practice, excluding the hypothesis of carcinogenesis.

## 1. Introduction

In 2021, Ozempic (semaglutide) was one of the most prescribed medications in the United States [1], with over 9 million prescriptions in the final quarter of 2022 [2], with the possibility of reaching as much as 24 million prescriptions by 2035 in the USA alone [3]. The drug has demonstrated significant utility in managing type 2 diabetes and obesity, thanks to its action as a glucagon-like peptide-1 (GLP-1) receptor agonist (RA) [4,5,6]. Semaglutide operates by enhancing insulin secretion and suppressing glucagon release, which in turn helps control blood sugar levels and supports weight loss [7,8]. The majority of semaglutide prescriptions (70%) were for the injection form at a concentration of 2 mg per 1.5 mL, showcasing its preferred method of administration for its efficacy in glycemic control and weight management [9,10].

The side effects most commonly associated with semaglutide include gastrointestinal issues such as nausea, vomiting, diarrhea, abdominal pain, and constipation. Moreover, these effects may be exacerbated when semaglutide is taken in association with other substances [11,12,13,14]. However, the rise in GLP1-RAs use has necessitated a thorough evaluation of its safety profile, particularly concerning its carcinogenic potential [15]. The Food and Drug Administration (FDA) has issued a boxed warning for semaglutide, based on animal studies indicating a risk for thyroid C-cell tumors [16]. Recent rodent studies indicate that long-term exposure to liraglutide, a GLP-1 receptor agonist, can lead to thyroid C-cell hyperplasia and tumors due to a GLP-1 receptor-mediated mechanism in rodents, which contrasts with the lack of similar findings in primates [17]. Although these findings raise concerns, the direct applicability to human risk remains uncertain, underscoring the importance of conducting additional studies to assess the carcinogenic effect, especially the thyroid carcinogenic risk associated with semaglutide, given the in vitro study findings [18,19].

Semaglutide was approved for medical use in the US in 2017, and its indications have expanded over the years to include not just type 2 diabetes management but also long-term weight management for adults with obesity or who are overweight and have at least one weight-related comorbidity [20]. In recent developments, the FDA further expanded semaglutide’s indication to include reducing the risk of cardiovascular death, heart attack, and stroke in adults with cardiovascular disease who are either obese or overweight.

Given semaglutide’s broadening scope of application, understanding its dosages, administration, and potential side effects is essential for healthcare professionals. Its growing prescription rates reflect its significance in the treatment landscape for diabetes and obesity, positioning it as a critical focus for ongoing research and patient care strategies. In light of these considerations, this systematic review proposed aims to critically analyze all available data concerning the incidence of thyroid cancer among patients treated with semaglutide and identify the most frequent and serious adverse events. The current study’s focus solely on semaglutide, among various GLP-1 receptor agonists, and specifically on thyroid cancer, is guided by the imperative to elucidate the nuanced risk profiles attributed to different therapeutic agents within the same class. This approach is grounded in the understanding that despite sharing a therapeutic class, individual GLP-1 RAs can exhibit diverse biological effects and safety profiles due to their distinct molecular structures and mechanisms of action [1,4,5,6].

## 2. Materials and Methods

### 2.1. Protocol and Registration

This study employed a detailed search strategy across three electronic databases to examine the existing literature on the thyroid carcinogenic risk associated with GLP-1 receptor agonist semaglutide (Ozempic) therapy. Databases including PubMed, Scopus, and Embase were systematically searched for literature published up until December 2023, to include the most current studies available on this critical topic.

The search strategy utilized a wide range of keywords and phrases relevant to the study’s aims, centering on the assessment of thyroid cancer risk in patients undergoing Semaglutide therapy. Key search terms included: “Semaglutide”, “Ozempic”, “thyroid cancer”, “thyroid neoplasms”, “GLP-1 receptor agonists”, “carcinogenic risk”, “thyroid C-cell tumors”, “medullary thyroid carcinoma”, “endocrine neoplasms”, “antidiabetic agents and cancer risk”, “GLP-1 safety”, “GLP-1 complications”, “semaglutide complications”, and “semaglutide adverse effects”.

Boolean operators (AND, OR, NOT) were strategically employed to refine and link the search terms effectively. The search string was constructed as follows: (“Semaglutide” [MeSH] OR “Ozempic”) AND (“Thyroid Neoplasms” [MeSH] OR “thyroid cancer” OR “thyroid C-cell tumors” OR “medullary thyroid carcinoma”) AND (“Glucagon-Like Peptide 1 Receptor Agonists” [MeSH] OR “carcinogenic risk” OR “endocrine neoplasms”) AND (“Carcinogenesis” [MeSH] OR “antidiabetic agents and cancer risk” OR “safety of GLP-1” OR “semaglutide adverse effects” OR “semaglutide adverse reactions” OR “semaglutide complications” OR “neoplastic risk”) AND (“Pharmacovigilance” [MeSH] OR “Drug-Related Side Effects and Adverse Reactions” [MeSH]).

Adhering to the Preferred Reporting Items for Systematic Reviews and Meta-Analyses (PRISMA) guidelines [21], this systematic review protocol ensures a structured, transparent, and reproducible methodology. To promote transparency and facilitate open access to our research process and findings, the review has been registered with the Open Science Framework (OSF) with the registration code osf.io/5kqwh.

### 2.2. Inclusion and Exclusion Criteria

The eligibility criteria were carefully formulated to identify studies that provide insights into the thyroid carcinogenic risk associated with the use of GLP-1RA semaglutide (Ozempic) therapy. The review considered the following inclusion criteria: (1) Study population: studies must involve patients who were undergoing or had undergone treatment with semaglutide. There was no age restriction applied, considering the broad application of semaglutide in adult populations for T2DM and obesity management. (2) Focus on thyroid carcinogenic risk: research specifically needed to mention the incidence of thyroid cancer risk among the study outcomes, or to report to malignancies during the follow-up period. This included studies assessing thyroid C-cell tumors, medullary thyroid carcinoma, and other thyroid neoplasms potentially linked to semaglutide use. (3) Types of studies: a wide range of study designs was included, such as randomized controlled trials (RCTs), observational studies, clinical trials, cohort studies, case-control studies, and cross-sectional studies. Studies were required to provide clear and detailed methodologies regarding the assessment of thyroid carcinogenic risk associated with semaglutide. (4) Outcome measures: studies were considered for analysis if they reported all complications, adverse events, and serious events. This could include patient-reported symptoms and clinical diagnosis of severe complications. (5) Diabetes and obesity: studies focusing on patients with diabetes and obesity only to avoid the potential confounding effect of other pathologies on the risk of cancer development or other complications. (6) Language: only peer-reviewed articles published in English were included to ensure the feasibility of thorough review and analysis.

The exclusion criteria comprised the following: (1) Non-human studies: research not involving human participants, such as in vitro or animal model studies, was excluded to focus solely on human patient experiences and outcomes. (2) Broad medication focus: studies not specifically examining patients treated with semaglutide, the use of other formulations of GLP1-RAs, or those that did not differentiate the impact of semaglutide from other GLP-1RAs or antidiabetic medications were excluded. (3) Lack of specific outcomes: studies that did not provide clear, quantifiable outcomes related to thyroid carcinogenic incidence, or lacked sufficient detail about the number of patients that were diagnosed with cancer during the study period were excluded. (4) Grey literature: to maintain the credibility and reliability of the data included in the review, grey literature, including non-peer-reviewed articles, preprints, conference proceedings, general reviews, commentaries, and editorials, was excluded.

### 2.3. Data Collection Process

**Figure 1:**
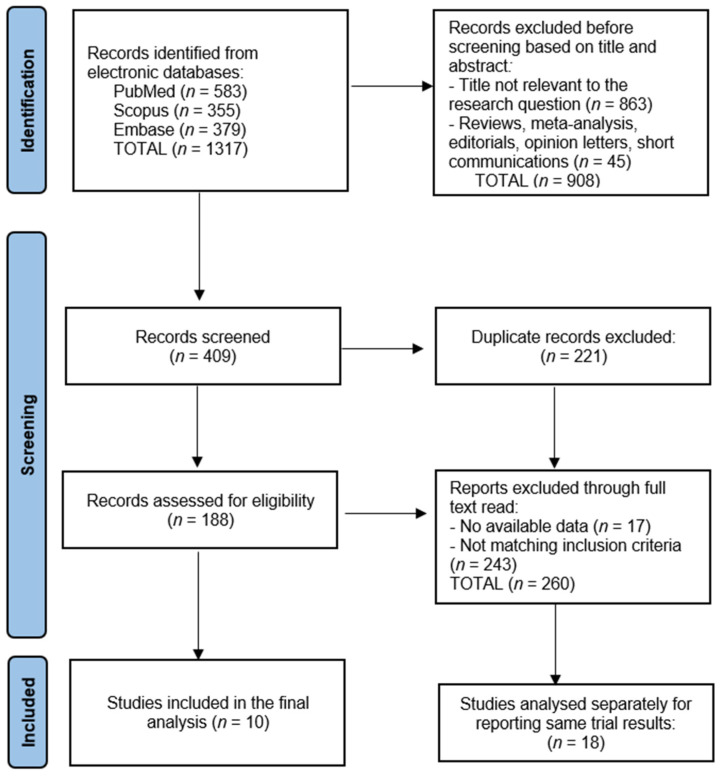
PRISMA flow diagram.

The data collection process for this systematic review commenced with the removal of 221 duplicate entries, followed by the screening of 409 abstracts by two independent reviewers to assess each study’s relevance, based on predefined inclusion and exclusion criteria. Discrepancies between the reviewers were resolved through discussion or, if necessary, consultation with a third reviewer to achieve consensus. The initial database search resulted in a number of 1317 articles, from which 28 relevant studies were identified as eligible for inclusion in the final study, of which 18 studies were analyzed separately for reporting same trial results, as presented in Figure 1.

### 2.4. Risk of Bias and Quality Assessment

For the systematic assessment of study quality and determination of risk of bias within the included studies, our review employed a dual approach, integrating both qualitative and quantitative evaluation methods. Initially, the quality of observational studies was evaluated using the Newcastle–Ottawa Scale [22]. To ensure the objectivity and reproducibility of our quality assessment process, each study was independently evaluated by two researchers. Discrepancies in quality assessment scores were resolved through discussion, or if necessary, consultation with a third researcher.

## 3. Results

### 3.1. Study Characteristics

This systematic review analyzed a total of 10 studies focused on assessing the thyroid carcinogenic risk associated with the GLP-1 receptor agonist semaglutide (Ozempic) therapy. These studies were multinational and spanned from 2016 to 2022, employing a randomized controlled trial (RCT) design to ensure high-quality evidence (Ahrén et al. [23], Buse et al. [24], Husain et al. [25], Marso et al. [26], Pratley et al. [27], Rosenstock et al. [28], Sorli et al. [29], Wadden et al. [30], Wilding et al. [31], Yamada et al. [32]), as presented in Table 1. The inclusion of phase II/IIIa to phase III trials, both double-blind and open-label extensions, highlighted the depth of the investigation into semaglutide’s safety profile.

### 3.2. Patients’ Characteristics

The results from Table 2 encompass a considerable cohort of 14,550 patients, out of which 7830 were treated with semaglutide across various clinical settings. The sample sizes ranged widely, from as few as 98 participants in the study by Buse et al. [24] to as many as 1648 in the study by Marso et al. [26]. The analysis uncovered an average age of approximately 57.3 years among the participants, while the ages of participants across these studies ranged from an average of 46 years in the studies by Wadden et al. [30] and Wilding et al. [31] to 66 years in Husain et al. [25]. The gender distribution within these studies showed a slight female predominance, with an average of 47.2% of the participants being male, ranging from 22.6% in Wadden et al. [30] to a notably higher 76.3% in Yamada et al. [32], indicating a diverse demographic engagement in semaglutide clinical trials.

The comparison groups in these studies were primarily either placebo or another antidiabetic medication, such as Sitagliptin or Liraglutide. In terms of disease duration, patients had been managing diabetes for an average of approximately 9.7 years before participating in these studies. Disease duration among participants treated with semaglutide ranged from 4.18 years in Sorli et al. [29] to 14.7 years in Husain et al. [25].

### 3.3. Weight and Glucose Levels

Semaglutide demonstrated therapeutic impact across varied treatment timelines and doses. Follow-up durations extended from 30 weeks in Sorli et al. [29] to an integrated period of 120 weeks in Wilding et al. [31], providing a comprehensive view of semaglutide’s long-term efficacy and safety. The administration of semaglutide spanned from lower dosages of 0.5 mg to higher, targeted doses such as the 2.4 mg subcutaneous injection, employed in studies by Wadden et al. [30] and Wilding et al. [31], reflecting the adaptability of semaglutide’s dosing to patient needs and clinical objectives.

Significant reductions in HbA1c were reported, ranging from −0.2% in the study by Buse et al. [24] with a flexible dose of oral semaglutide, to −1.7% for the 14 mg dose in the study by Yamada et al. [32], demonstrating semaglutide’s robust glycemic control capabilities across various dosages. These reductions in HbA1c levels were accompanied by notable weight loss, with changes from −1.9 kg in the study by Ahrén et al. [23] with Sitagliptin as the comparison group, to an impressive −16.0% of body weight loss reported in Wadden et al. [30] with a 2.4 mg weekly dose of subcutaneous semaglutide, as presented in Table 3.

### 3.4. Thyroid Cancer Incidence and Complications

In Table 4, the evaluation of study outcomes and thyroid cancer incidence provided insight into the safety profile of semaglutide. Across the 10 studies, thyroid cancer incidence was notably low, with a few isolated cases of papillary thyroid cancer and medullary thyroid cancer reported, each constituting less than 1% within the respective study groups [23,25,30,32], suggesting no significant risk for thyroid cancer associated with semaglutide use when considering the large sample sizes.

**Figure 2:**
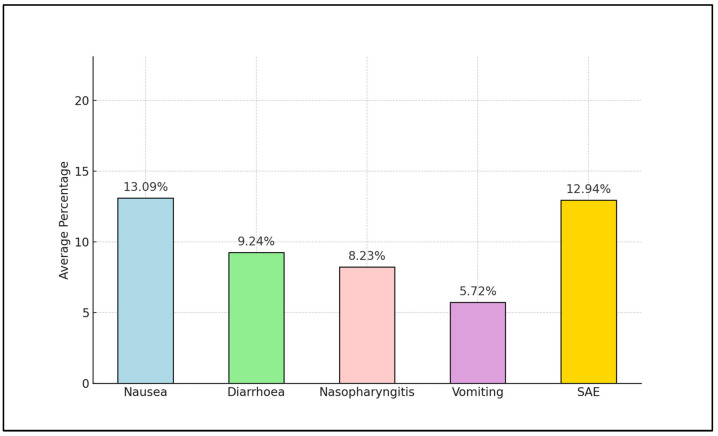
Aggregate mean values with adverse events and complications across all studies after semaglutide use.

Adverse events commonly reported were gastrointestinal in nature, such as nausea and diarrhea, with occurrences ranging from 2.05% to 19.95% for nausea [24,26] and 1.4% to 13% for diarrhea [23,29]. Nasopharyngitis and vomiting were also reported, though less frequently, with nasopharyngitis averaging 8.23% and vomiting at 5.72%, based on the aggregate values presented in Figure 1. Major side complications were predominantly serious adverse events, with rates varying from 7% to 25.2% [26,29]. Severe hypoglycemia and pancreatitis were reported as well, albeit less commonly [23]. Figure 2 graphically depicts the average percentage of adverse events reported across the studies, highlighting nausea as the most common adverse event, with an average of 13.09%; followed by diarrhea at 9.24%; and serious adverse events at 12.94%.

## 4. Discussion

### 4.1. Summary of Evidence

In this systematic review, the collective data from rigorous randomized controlled trials provided valuable evidence to address concerns regarding the thyroid carcinogenic risk associated with semaglutide (Ozempic) therapy. Given the duration of the trials, some with follow-up periods extending beyond two years, the incidence of thyroid cancer was remarkably low, with reported cases being isolated and representing less than 1% of study populations. This extended follow-up period is critical in assessing long-term risks, such as cancer development, allowing for some confidence in ruling out a significant association between semaglutide and thyroid cancer. It is essential to recognize the strength of randomized control trials in establishing causality, and these findings contribute substantively to the safety profile of semaglutide in this context.

The reported adverse events were aligned with the known side effect profile of GLP-1 receptor agonists. Gastrointestinal events, such as nausea and diarrhea, were the most common, yet they did not detract from the overall benefits observed in glycemic control and weight reduction. The consistency of these side effects across studies accentuates the need for patient education and clinical monitoring but does not diminish the clinical utility of semaglutide.

In the assessment of semaglutide’s safety profile from the studies included in the final analysis, adverse events primarily related to gastrointestinal symptoms were observed as most common, along with other side effects. Nasopharyngitis showed a mean prevalence of approximately 8.23%, with occurrences ranging from 5% in the study by Sorli et al. [29] to 10.2% in Buse’s study [24]. Vomiting had a mean prevalence of nearly 5.97%, with the range extending from 1.5% in the study by Husain et al. [25] to 9% in Ahren’s study [23]. An increase in lipase levels was noted, with a mean of 6.5%, ranging from 5% in Sorli et al.s’ study [29] to 8% in the study by Ahren et al. (SUSTAIN 2 trial) [23], reflecting a noteworthy side effect. Headaches were reported, with a mean prevalence of 7.92%, spanning from 6.5% also in the SUSTAIN 2 trial [23] to 9.5% in the SUSTAIN 1 [29]. Decreased appetite was consistently reported at 7% in Ahren’s study [23]. Influenza symptoms had a mean prevalence of 5.23%, with reports ranging from 4.5% in the SUSTAIN 2 trial [23] to 5.95% in the PIONEER 7 trial [24]. Dyspepsia averaged a prevalence of 5.18%, with occurrences ranging from 4.5% in Sorli’s study [29] to 5.55% in the study by Buse et al. [24]. Lastly, constipation had a mean prevalence of 6.91%, with the range extending from 4.65% in the PIONEER 7 study [24] to 13% in the study by Yamada et al. [32], indicating a variable but significant gastrointestinal adverse event. These analyses illustrate the spectrum of adverse events associated with semaglutide use, underlining the importance of monitoring for gastrointestinal and other side effects in patients treated with this medication.

Other potential studies were excluded from the final analysis due to a lack of mention of cancer incidence or thyroid cancer incidence in their study population, their focus on studying outcomes from the same clinical trials, or their lack of focus on patients with diabetes mellitus and obesity [25,33,34,35,36,37,38,39,40,41,42,43,44,45,46,47,48,49]. For instance, studies such as those by Aroda VR et al. [33] and Zinman B et al. [35] were omitted because they did not focus on or mention thyroid cancer incidence, despite discussing semaglutide’s effects in diabetes management. Similarly, trials such as Davies M et al. [36], Loomba R et al. [37], and Weghuber D et al. [41] did not center on diabetes or obesity as primary conditions, thereby not meeting the inclusion criteria, which focused on these specific chronic diseases. Furthermore, studies such as those by Kellerer M et al. [47] and Ji L et al. [48] were not included, as neither discussed the same trial results.

The PIONEER trial series, such as PIONEER 1 by Aroda VR et al. [33] and PIONEER 5 by Mosenzon O et al. [34], were omitted because they either reported on the same patient groups as the included studies without mentioning thyroid cancer incidence or investigated patient subgroups outside this review’s scope. Similarly, the SUSTAIN and STEP series, including trials such as SUSTAIN 11 by Kellerer M et al. [47] and STEP TEENS by Weghuber D et al. [41], were not considered, as they either overlapped with included studies or did not report on this review’s primary outcomes.

Regarding the risk of other cancer types, the study by Nagendra et al. [50] did not identify any significant risk in any types of neoplasms associated with semaglutide use, with an overall odds ratio of 0.95 (95% CI: 0.62–1.45). Similarly, the risk of pancreatic cancer, which was also hypothesized to be associated with GLP-1 medication, did not show any significant increase, with an OR of 0.25 (95% CI: 0.03–2.24). Besides the risk of cancer, an extended analysis of severe complications revealed that a total of 9228 patients took semaglutide across 29 studies [23,24,25,26,27,28,29,30,31,32,33,34,35,36,37,38,39,40,41,42,43,44,45,46,47,48,49,50]. Within this cohort, there were 320 instances of severe hypoglycemia, translating to a proportion of approximately 3.47%. Acute kidney injury (AKI) was reported in 18 cases, constituting about 0.20% of the semaglutide patients. Furthermore, 22 cases of pancreatitis were documented, representing roughly 0.24% of the patients treated with semaglutide.

In a detailed examination of studies involving patients treated with sitagliptin [23,28,48] and liraglutide [27,43,46] as comparison groups for semaglutide, distinct outcomes have been observed. The sitagliptin group comprised 1518 patients, within which there were 62 reported cases of severe hypoglycemia, resulting in a proportion of approximately 4.08%. Additionally, this group experienced a lower incidence of acute kidney injury (AKI) and pancreatitis, with only three cases (0.20%) and one case (0.07%), respectively. In comparison, the liraglutide cohort included 849 patients. This group had 21 cases of severe hypoglycemia, translating to a proportion of about 2.47%. The occurrences of AKI were notably rare, with only one reported case (0.12%), while pancreatitis cases were somewhat higher, at four, representing 0.47% of the liraglutide-treated patients.

Another study investigating the carcinogenic risks associated with GLP-1s, including semaglutide, liraglutide, exenatide, and dulaglutide, encompassed a total of 69,909 patients across twenty-six trials that reported at least one incident case of thyroid cancer [51]. Within these findings, 86 cases of thyroid cancer were identified (60 in the GLP-1RA arm and 26 in the comparator arms). Of these, 25 cases (19 in the GLP-1RA arm versus 6 in comparator arms) were identified as papillary thyroid carcinomas (PTCs) and three as medullary thyroid carcinomas, with two associated with GLP-1RAs and one with comparators.

Bezin et al.’s study [52] on the risk of thyroid cancer associated with GLP-1 receptor agonists diverges from our findings regarding semaglutide, suggesting a potential increased risk for thyroid cancer. Involving 2562 patients diagnosed with thyroid cancer and matched with 45,184 control subjects, this extensive research utilized the French national healthcare insurance system database, focusing on T2DM patients treated with second-line antidiabetes drugs from 2006 to 2018. Notably, the study found that the use of GLP-1 RAs for a duration of 1–3 years was associated with a heightened risk of all thyroid cancers, presenting an adjusted hazard ratio of 1.58 (95% CI 1.27–1.95) and an adjusted HR of 1.78 (95% CI 1.04–3.05), specifically for medullary thyroid cancer. These findings stand in contrast to prior data, showing no significant risk of thyroid cancer with semaglutide use, suggesting that differences in study design, population, and possibly the formulations of GLP-1 RAs used could contribute to these varying outcomes.

In light of the concern surrounding the implications of GLP-1RAs on thyroid health, existing studies found an important association when involving obese patients. Schmid et al. [53] uncovered that obesity is linked to a significantly higher risk of thyroid cancer, with overweight individuals facing a 25% increased risk and obese individuals a 55% increased risk compared to their normal-weight counterparts. Additionally, for every 5-unit increase in BMI, the risk of thyroid cancer escalates by 30%. Conversely, Hu et al.’s investigation into GLP-1RAs presented a nuanced picture, indicating an association between GLP-1RA use and an increased risk of general thyroid disorders (RR 1.28, 95% CI 1.03–1.60), but not specifically thyroid cancer (RR 1.30, 95% CI 0.86–1.97) [54]. These findings underscore the complexity of the relationship between obesity, GLP-1RA use, and thyroid health, highlighting the need for careful consideration of obesity as a factor in thyroid cancer risk assessment in the context of GLP-1RA treatment.

Although a dose-dependent risk assessment for thyroid cancer was not available, one study found that administering a dose of liraglutide eight times higher than the highest approved dose for humans was associated with a potential carcinogenic factor, indicating that GLP-1RAs may have a dose-dependent effect on cell proliferation in thyroid dysplastic or premalignant lesions [55]. This evidence underscores the importance of closely monitoring the dose-dependent implications of GLP-1RAs on thyroid cellular changes, highlighting the critical need for caution in their clinical application.

Nevertheless, according to the European Medicines Agency’s (EMA) Pharmacovigilance Risk Assessment Committee (PRAC) findings from October 2023, there is no evidence to suggest a causal relationship between GLP-1 receptor agonists (including exenatide, liraglutide, dulaglutide, semaglutide, and lixisenatide) and thyroid cancer [56]. This conclusion, drawn after reviewing extensive observational and cumulative data, indicates that no amendments to the current product information for these medications are necessary at this time. The PRAC emphasizes the need for ongoing surveillance and reporting on this issue through periodic safety update reports.

The clinical utility of this study lies in its consolidation of data from multiple randomized controlled trials, elucidating the thyroid cancer risk associated with semaglutide, an area of concern for clinicians and patients, given the drug’s increasing usage for diabetes and weight management. The study’s novelty lies in the inclusion of long-term follow-up data from all existing studies up to December 2023, which provides reassurance regarding the safety of semaglutide in relation to thyroid malignancies, a problem not extensively explored in previous research. Ultimately, while no research can conclusively rule out all risks, especially with rare outcomes such as thyroid cancer, the data presented here suggest that the risk is, at a minimum, very low. This review, therefore, provides a solid foundation for clinicians to make informed decisions regarding semaglutide use, weighing the substantial benefits against the potential but seemingly low risk of thyroid cancer.

### 4.2. Limitations

The study’s limitations are inherent in the nature and duration of the clinical trials analyzed. Although randomized trials are robust in design, the post-marketing period and the real-world use of semaglutide may reveal further insights into its safety profile. Thus, ongoing surveillance is essential to monitor for delayed adverse outcomes, which this study could not account for. Furthermore, the studies exhibited a high degree of variability, which made it impossible to conduct a funnel plot analysis for assessing publication bias. Additionally, the reporting of study outcomes lacked the uniformity necessary to conduct a meta-analysis effectively.

## 5. Conclusions

This systematic review offers important insights into the safety records of semaglutide (Ozempic), particularly highlighting gastrointestinal side effects and its negligible risk towards thyroid cancer. Although gastrointestinal complaints are frequently reported, semaglutide continues to be an effective treatment alternative for diabetes and obesity management. The extensive analysis and description of several adverse events that affect more than 10% of patients emphasizes the need for vigilant monitoring and effective management of these issues in a clinical setting. Although other significant risks can be considered negligible based on this study results on semaglutide, other GLP-1 RAs can determine different outcomes.

## Figures and Tables

**Table 1:** Study characteristics.

Study & Author	Country	YOP	Study Design	Quality of Evidence
1 [23] Ahrén et al.	Multi-national	2017	RCT SUSTAIN 2 DB—Phase IIIa	High
2 [24] Buse et al.	Multi-national	2020	RCT PIONEER 7 Open-label extension	High
3 [25] Husain et al.	Multi-national	2019	RCT PIONEER 6 Phase IIIa	High
4 [26] Marso et al.	Multi-national	2016	RCT SUSTAIN 6 DB—Phase III	High
5 [27] Pratley et al.	Multi-national	2019	RCT PIONEER 4 Phase IIIa	High
6 [28] Rosenstock et al.	Multi-national	2019	RCT PIONEER 3 DB—Phase IIIa	High
7 [29] Sorli et al.	Multi-national	2017	RCT SUSTAIN 1 DB—Phase IIIa	High
8 [30] Wadden et al.	United States	2021	RCT Phase III	High
9 [31] Wilding et al.	Multi-national	2022	RCT STEP 1 DB	High
10 [32] Yamada et al.	Multi-national	2020	RCT PIONEER 9 DB—Phase II/IIIa	High

YOP—year of publication; RCT—randomized controlled trial; DB—double blind.

**Table 2:** Patients’ characteristics.

Study Number	Sample Size	Age (Years)	Gender (Male)	Comparison Group	Disease Duration
1 [23] Ahrén et al.	Semaglutide 0.5 mg: 409, Semaglutide 1.0 mg: 409	Semaglutide 0.5 mg: 54.8, Semaglutide 1.0 mg: 56.0, Sitagliptin: 54.6	Semaglutide 0.5 mg: 51%, Semaglutide 1.0 mg: 50%, Sitagliptin: 51%	Sitagliptin 100 mg: 407	Semaglutide 0.5 mg: 6.4 years, Semaglutide 1.0 mg: 6.7 years, Sitagliptin: 6.6 years
2 [24] Buse et al.	Oral Semaglutide: 100	Oral Semaglutide: 58, Sitagliptin: 58	Oral Semaglutide: 43.0%, Sitagliptin: 43.9%	Sitagliptin: 98	Oral Semaglutide: 8.1 years, Sitagliptin: 9.6 years
3 [25] Husain et al.	Oral Semaglutide: 1591	Mean: 66	68.1%	Placebo: 1592	Semaglutide: 14.7 years, Placebo: 15.1 years
4 [26] Marso et al.	Subcutaneous Semaglutide: 1648 (0.5 mg: 826, 1.0 mg: 822)	Semaglutide 0.5 mg: 64.6, Semaglutide 1.0 mg: 64.7	34.1%	Placebo: 1649	Semaglutide: 14.2 years, Placebo: 13.6 years
5 [27] Pratley et al.	Oral Semaglutide 14 mg: 285, Liraglutide 1.8 mg: 284	Semaglutide: 56, Liraglutide: 56	Oral Semaglutide: 52%, Liraglutide: 52%	Placebo: 142	Semaglutide: 7.8 years, Liraglutide: 7.3 years, Placebo: 7.8 years
6 [28] Rosenstock et al.	Oral Semaglutide: 1396 (3 mg: 466, 7 mg: 466, 14 mg: 465), Sitagliptin: 467	Oral Semaglutide: Mean 58, Sitagliptin: Mean 58	Oral Semaglutide: 53.1%, Sitagliptin: 51.0%	Sitagliptin 100 mg: 467	Oral Semaglutide: Mean 8.6 years, Sitagliptin: Mean 8.8 years
7 [29] Sorli et al.	Semaglutide: 258	Semaglutide: 53.7, Placebo: 53.9	Semaglutide: 54%, Placebo: 54%	Placebo: 129	Semaglutide: 4.18 years, Placebo: 4.06 years
8 [30] Wadden et al.	Semaglutide: 407	Mean: 46	Semaglutide: 22.6%, Placebo: 11.8%	Placebo: 204	NR
9 [31] Wilding et al.	Semaglutide: 1306	Mean: 46	Semaglutide: 26.9%, Placebo: 24.0%	Placebo: 655	NR
10 [32] Yamada et al.	Oral Semaglutide 3 mg: 49, 7 mg: 49, 14 mg: 48	Semaglutide: 60, Placebo: 59, Liraglutide: 59	Semaglutide: 76.3%, Placebo: 82%, Liraglutide: 81%	Placebo: 49, Liraglutide: 48	Semaglutide: 7.6 years, Placebo: 8.4 years, Liraglutide 6.7

NR—not reported.

**Table 3:** Analysis of follow-up duration, medication dosage and changes in glucose levels and weight.

Study Number	Follow-Up	Dose	HbA1c/Fasting Glucose	Weight (Initial, Weight Change)
1 [23] Ahrén et al.	56 weeks	Semaglutide: 0.5 mg, 1.0 mg; Sitagliptin: 100 mg	Semaglutide: −1.3% (0.5 mg), −1.6% (1.0 mg); Sitagliptin: −0.5%	Initial: 89.5 kg; Change: −4.3 kg (Semaglutide 0.5 mg), −6.1 kg (Semaglutide 1.0 mg), −1.9 kg (Sitagliptin)
2 [24] Buse et al.	52 weeks	Oral semaglutide: flexible dose (0.5–1 mg); sitagliptin: 100 mg	Oral Semaglutide: −0.2%; Sitagliptin: +0.1%	Initial: 85.8 kg (Oral Semaglutide), 86.9 kg (Sitagliptin); Change: −2.4 kg (Oral Semaglutide), −0.9 kg (Sitagliptin)
3 [25] Husain et al.	15.9 months	Oral semaglutide: 14 mg target dose	Baseline: 8.2 ± 1.6% HbA1c; decrease: oral semaglutide −1.0%, placebo −0.3%	Initial: 90.9 ± 21.2 kg; Change: −4.2 kg (Oral Semaglutide)
4 [26] Marso et al.	104 weeks	Semaglutide subcutaneous: 0.5 mg, 1.0 mg	Baseline HbA1c 8.7%; reduction: −1.1% (0.5 mg), −1.4% (1.0 mg)	Initial: 92.1 kg; Change: −3.6 kg (0.5 mg), −4.9 kg (1.0 mg)
5 [27] Pratley et al.	52 weeks	Oral semaglutide (escalated to 14 mg), liraglutide (escalated to 1.8 mg), placebo	HbA1c: Oral semaglutide: −1.2%, liraglutide: −1.1%, placebo: −0.2%	Initial: 94.0 kg; Change: Oral semaglutide: −4.4 kg, Liraglutide: −3.1 kg, Placebo: −0.5 kg
6 [28] Rosenstock et al.	78 weeks	Oral semaglutide (3 mg, 7 mg, 14 mg), sitagliptin 100 mg	Oral semaglutide: decrease in HbA1c −0.6% (3 mg), −1.0% (7 mg), −1.3% (14 mg); sitagliptin: decrease in HbA1c −0.8%	Oral Semaglutide: Initial 91.2 kg, Weight change −1.2 kg (3 mg), −2.2 kg (7 mg), −3.1 kg (14 mg), Sitagliptin: Initial 90.9 kg, Weight change −0.6 kg
7 [29] Sorli et al.	30 weeks	Semaglutide (0.5 mg, 1.0 mg), placebo	Semaglutide: −1.5% (1.0 mg), −1.45% (0.5 mg) vs. placebo: −0.02%	Semaglutide: −4.53 kg (1.0 mg), −3.73 kg (0.5 mg) vs. Placebo: −0.98 kg
8 [30] Wadden et al.	68 weeks	Semaglutide subcutaneous 2.4 mg	Fasting plasma glucose: semaglutide (−6.73 mg/dL), placebo (−0.65 mg/dL)	Weight loss: Semaglutide:(−16.0%), Placebo: (−5.7%)
9 [31] Wilding et al.	68 weeks (main phase) + 52 weeks (extension)	Semaglutide subcutaneous 2.4 mg once weekly	Reversion to normal blood glucose levels at 120 weeks: semaglutide (43.3%), placebo (34.0%)	By week 68: Semaglutide: 17.3%, Placebo: 2.0%. Regain by week 120: Semaglutide: 11.6%, Placebo: 1.9%. Net loss from baseline to week 120: Semaglutide: 5.6%, Placebo: 0.1%
10 [32] Yamada et al.	52 weeks	Oral semaglutide (3 mg, 7 mg, 14 mg), liraglutide 0.9 mg	Baseline HbA1c: 8.3% across all groups. Significant reductions compared to placebo: −1.1% for 3 mg, −1.5% for 7 mg, and −1.7% for 14 mg	Baseline weight 71.1 kg. Significantly more patients achieved a weight loss reduction of 5.0% or greater with oral semaglutide 14 mg than those who received placebo or liraglutide at both weeks 26 and 52

NR—not reported.

**Table 4:** Evaluation of study outcomes and thyroid cancer incidence.

Risk Factors	Thyroid Cancer	Adverse Events	Major Side Complications	Conclusions
1 [23] Ahrén et al.	PTC: 1 patient (<1%) in the 1.0 mg group OR: 1.50–NS	Nausea: 18%, diarrhea: 13%, nasopharyngitis: 9.5%, vomiting: 9%, lipase increased: 8%, headache: 6.5%, decreased appetite: 7%, influenza: 4.5%, dyspepsia: 5.5%, constipation: 5%	Severe hypoglycemia: 33, pancreatitis: 2	Once-weekly semaglutide superior to Sitagliptin in glycemic control and weight reduction for diabetes patients on metformin, thiazolidinediones, or both. Safety profile similar to other GLP-1 receptor agonists. No significant risk for thyroid cancer.
2 [24] Buse et al.	Thyroid cancer: 0%	Nausea: 19.95%, nasopharyngitis: 10.2%, diarrhea: 10.75%, headache: 7.75%, abdominal pain, upper: 6.95%, dyspepsia: 5.55%, vomiting: 7.05%, upper respiratory tract infection: 4.05%, arthralgia: 4.85%, back pain: 4.85%, influenza: 5.95%, gastroenteritis: 4.05%, constipation: 4.65%	SAE: 9.1% for oral semaglutide, 8.0% after sitagliptin switched to semaglutide	Long-term oral semaglutide treatment maintained HbA1c reductions with additional body weight reductions. Switching from sitagliptin to oral semaglutide maintained HbA1c reductions with potential for additional weight loss. No significant risk for thyroid cancer.
3 [25] Husain et al.	MTC: 1 patient (<1%)	Nausea: 2.9% (oral semaglutide group), 0.5% (placebo group); Vomiting: 1.5% (oral semaglutide), 0.3% (placebo); Diarrhea: 1.4% (oral semaglutide), 0.4% (placebo)	SAE: 18.9% (oral semaglutide) vs. 22.5% (placebo). Deaths lower in the oral semaglutide group (1.4%) compared to placebo (2.8%)	Cardiovascular risk profile of oral semaglutide not inferior to placebo in type 2 diabetes patients. No significant risk for thyroid cancer.
4 [26] Marso et al.	MTC: 0%	Adverse event leading to discontinuation: 13%, nausea: 3.4%, vomiting: 2.25%, diarrhea: 2.05%, gastrointestinal disorder: 51.5%, cardiac disorder: 19.55%, atrial fibrillation: 3.05%	SAE: 24.2% (semaglutide 0.5 mg), 25.2% (semaglutide 1.0 mg), placebo: 26.2% (0.5 mg) vs. 23.5% (1.0 mg)	Semaglutide significantly reduced primary composite cardiovascular outcome compared to placebo in high cardiovascular risk type 2 diabetes patients. No significant risk for thyroid cancer.
5 [27] Pratley et al.	Thyroid cancer: 0%	GI adverse events: oral semaglutide (80%), liraglutide (74%), placebo (67%). Hypoglycemic episodes: oral semaglutide (1%), liraglutide (2%), placebo (2%)	Nausea: oral semaglutide (20%), liraglutide (18%). Diarrhea: oral semaglutide (15%), liraglutide (11%). Vomiting: oral semaglutide (9%). Early discontinuation due to adverse events: semaglutide (11%), liraglutide (9%), placebo (4%)	Oral semaglutide demonstrated non-inferiority to subcutaneous liraglutide and superiority to placebo in reducing HbA1c and body weight. The safety and tolerability profile was consistent with the GLP-1 receptor agonist class, predominantly gastrointestinal events. No significant risk for thyroid cancer.
6 [28] Rosenstock et al.	Thyroid cancer: 0%	Nausea: semaglutide (11–20%), sitagliptin (6.5%). Diarrhea: semaglutide (9.7–10.6%), sitagliptin (6.4%). Hypoglycemia: semaglutide 3 mg (4.9%), 7 mg (5.2%), 14 mg (7.7%); sitagliptin (8.4%)	Symptomatic hypoglycemia: 3 mg/d (4.9%), 7 mg/d (5.2%), and 14 mg/d (7.7%); and in the sitagliptin group (8.4%). SAE: semaglutide 3 mg (10.1%), 7 mg (8.0%), and 14 mg (8.6%)	Oral semaglutide at 7 mg and 14 mg resulted in significantly greater reductions in HbA1c compared to sitagliptin over 26 weeks, with the 3 mg dosage showing no significant benefit over sitagliptin. The safety profile was consistent with expectations for the class. No significant risk for thyroid cancer.
7 [29] Sorli et al.	Thyroid cancer: 0%	Nausea: 22%, diarrhea: 12%, headache: 9.5%, lipase increased: 5%, constipation: 5%, dyspepsia: 4.5%, nasopharyngitis: 5%, vomiting: 5.5%	SAE: semaglutide 0.5 mg (7%), 1.0 mg (6%), placebo (3%)	Semaglutide significantly improved HbA1c and body weight in treatment-naive patients with type 2 diabetes compared to placebo. The safety profile was consistent with the GLP-1 receptor agonist class, with no significant risk for thyroid cancer.
8 [30] Wadden et al.	PTC: 1 patient (<1%)	Gastrointestinal adverse events: semaglutide (82.8%) vs. placebo (63.2%). Discontinuation due to adverse events: semaglutide (3.4%) vs. placebo (0%)	SAE: semaglutide (9.1%), placebo (2.9%)	Semaglutide, combined with intensive behavioral therapy and initial low-calorie diet, led to a significantly greater weight loss compared to placebo over 68 weeks in adults with overweight or obesity, with no significant risk for thyroid cancer.
9 [31] Wilding et al.	Thyroid cancer: 0%	NR	NR	After the withdrawal of semaglutide and lifestyle intervention, participants regained a significant portion of the weight they had lost, highlighting the chronic nature of obesity and the need for ongoing treatment to maintain weight loss and health improvements. No significant risk for thyroid cancer.
10 [32] Yamada et al.	Thyroid cancer: 1 patient in the oral semaglutide 7 mg	Constipation: semaglutide (10–13%), placebo (6%), liraglutide (19%). Nausea: semaglutide (7.5%), placebo (8%), liraglutide (2%)	SAE: semaglutide all dosages (3.4%), placebo (6%), liraglutide (0%)	Oral semaglutide significantly reduces HbA1c and induces weight loss in a dose-dependent manner in patients with T2DM, with a safety profile consistent with GLP-1 receptor agonists. No significant risk for thyroid cancer.

NR—not reported; PTC—papillary thyroid cancer; MTC—medullary thyroid cancer; OR—odds ratio; NS—not significant.

## Data Availability

Not applicable.
